# Significant Associations of lncRNA H19 Genotypes with Susceptibility to Childhood Leukemia in Taiwan

**DOI:** 10.3390/ph14030235

**Published:** 2021-03-08

**Authors:** Jen-Sheng Pei, Chao-Chun Chen, Wen-Shin Chang, Yun-Chi Wang, Jaw-Chyun Chen, Yu-Chen Hsiau, Pei-Chen Hsu, Yuan-Nian Hsu, Chia-Wen Tsai, Da-Tian Bau

**Affiliations:** 1 Department of Pediatrics, Taoyuan General Hospital, Ministry of Health and Welfare, Taoyuan 33004, Taiwan; pjs11pjs@gmail.com (J.-S.P.); yukino720701@gmail.com (C.-C.C.); zoehsu2813@gmail.com (P.-C.H.); 2 Graduate Institute of Biomedical Sciences, China Medical University, Taichung 404333, Taiwan; halittlemelon@hotmail.com (W.-S.C.); s1245624@gmail.com (Y.-C.W.); wenwen816@gmail.com (C.-W.T.); 3 Terry Fox Cancer Research Laboratory, Department of Medical Research, China Medical University Hospital, Taichung 404332, Taiwan; blanchehsiau@gmail.com; 4 Department of Medicinal Botanicals and Health Applications, Da-Yeh University, Changhua 515006, Taiwan; jawchyunchen@gmail.com; 5 Department of Family Medicine, Taoyuan General Hospital, Ministry of Health and Welfare, Taoyuan 33004, Taiwan; hsunian66@gmail.com; 6 Department of Bioinformatics and Medical Engineering, Asia University, Taichung 41354, Taiwan

**Keywords:** lncRNA, *H19*, SNP, childhood leukemia, genetic susceptibility, Taiwan

## Abstract

The purpose of our study was to investigate whether genetic variations in lncRNA *H19* were associated with susceptibility to childhood leukemia. Two hundred and sixty-six childhood leukemia patients and 266 healthy controls were enrolled in Taiwan, and two single nucleotide polymorphisms (SNPs), rs2839698 and rs217727, in *H19* were genotyped and analyzed. There was a significant difference in the genotypic distribution of rs2839698 between patients and healthy controls (*p* = 0.0277). Compared to the wild-type CC genotype, the heterozygous variant CT and homozygous variant TT genotypes were associated with significantly increased risks of childhood leukemia with an adjusted odd ratio (OR) of 1.46 (95% confidence interval (CI), 1.08–2.14, *p* = 0.0429) and 1.94 (95%CI, 1.15–3.31, *p* = 0.0169), respectively (*p*_for tread_ = 0.0277). The difference in allelic frequencies between childhood leukemia patients and controls was also significant (T versus C, adjusted OR = 1.53, 95%CI, 1.13–1.79, *p* = 0.0077). There were no significant differences in the genotypic and allelic distributions of rs217727 between cases and controls. Interestingly, the average level of *H19* rs2839698 was statistically significantly higher for patients with CT and TT genotypes than from those with the CC genotype (*p* < 0.0001). Our results indicate that *H19* SNP rs2839698, but not rs217727, may serve as a novel susceptibility marker for childhood leukemia.

## 1. Introduction

Acute lymphoblastic leukemia is the most common type of malignancy among children worldwide. The contributions of inherited genetic factors to the etiology of childhood leukemia have been reported by a few candidate gene association studies, but more remain to be found [1,2,3,4,5,6,7,8,9,10]. Long non-coding RNAs (lncRNAs) are defined as non-protein-coding transcripts [11]. Although the functions of most lncRNAs are still not well understood, the number of functionally characterized lncRNAs keeps increasing. LncRNAs play important roles in the regulation of gene expression at transcriptional and post-transcriptional level and are involved in development, differentiation, and human diseases [12,13,14,15,16,17]. In recent years, the contributions of lncRNAs to carcinogenesis have been documented and have attracted the interest of scientists [18,19,20].

*H19* is a lncRNA of 2.3 kb [21], which is known to be abundantly expressed in embryonal tissue and dramatically down-regulated after birth [22]. Abnormal over-expression of H19 has been noted in many types of cancer [23], and accumulating evidence indicated that H19 is involved in cancer initiation, progression, and metastasis [24]. Given the oncogenic roles of H19 and the functions of genetic variants in modulating the expression or structure of *H19*, increasing studies have been performed to examine the associations of single nucleotide polymorphisms (SNPs) in *H19* with genetic susceptibility to cancers [25,26,27]. Two SNPs, rs2839698 and rs217727, have been shown to be associated with the risks of different cancers, such as bladder cancer and breast cancer [25,26,27]. However, no study has been conducted in childhood leukemia. We hypothesize that *H19* rs2839698 and rs217727 SNPs may confer genetic susceptibility to childhood leukemia and thus conduct this case-control study to examine the association of these two SNPs with childhood leukemia in a Taiwan population.

## 2. Results

### 2.1. Comparisons of Basic Characters between the Case and Control Groups

The investigated population in this study contained 266 Taiwan childhood leukemia cases and 266 age- and gender-matched healthy children. The age was frequency-matched, and gender was one-on-one matched. The mean age ± standard deviation (SD) was 7.0 ± 4.4 for the cases and 8.3 ± 4.8 for the controls (*p* = 0.6483). There were 148 boys and 118 girls in cases and controls (Table 1).

### 2.2. The Relationships between H19 rs2839698 Polymorphism and Risk of Childhood Leukemia

Table 2 shows the genotypic frequencies of *H19* rs2839698 SNP in cases and controls. The genotypic distribution was consistent with the Hardy–Weinberg equilibrium (HWE) in the controls (*p* = 0.8781). The genotype distribution was significantly different between cases and controls (*p* = 0.0277). In multivariate logistic regression analysis, compared to the wild-type CC genotype, the heterozygous variant CT and homozygous variant TT genotypes were associated with significantly increased risks of childhood leukemia with an adjusted OR of 1.46 (95%CI, 1.08–2.14, *p* = 0.0429) and 1.94 (95%CI, 1.15–3.31, *p* = 0.0169), respectively. In the dominant model, individuals carrying the variant genotypes (CT+TT) had an elevated risk of childhood leukemia (adjusted OR = 1.68, 95%CI = 1.12–2.23, *p* = 0.0130, Table 2) compared to the CC genotype. In the allelic test, the “T” allele was associated with a significantly increased risk of childhood leukemia compared to the “C” allele (adjusted OR = 1.53, 95%CI = 1.13–1.79, *p* = 0.0077, Table 3).

### 2.3. The Relationships between H19 rs217727 Polymorphism and Risk of Childhood Leukemia

The genotypic frequencies of the *H19* rs217727 SNP among childhood leukemia patients and controls are shown in Table 4. The genotypic distribution of *H19* rs217727 polymorphism among the control group was consistent with HWE (*p* = 0.9610). There was no significant difference in genotypic distribution of the rs217727 SNP between childhood leukemia patients and controls (*p* = 0.9165). There were no altered risks of childhood leukemia associated with rs217727 in both genotypic (Table 4) and allelic tests (Table 5).

### 2.4. The Relationships between H19 rs2839698 Polymorphism with Clinical Features (Immunophenotypes, Risk Classification, and Survival Time)

The association between the *H19* rs2839698 genotype with immunophenotypes, risk classification, and survival time of childhood leukemia are shown in Table 6. No statistically significant correlation was observed between *H19* rs2839698 genotypic distributions and immunophenotypes (Table 6). Interestingly, the percentages of CT + TT genotypes of *H19* rs2839698 were statistically higher among the patients of in the high risk and very high risk groups, with an adjusted OR of 1.46 (95%CI = 1.04–1.87) and 1.38 (95%CI = 1.02–1.79), respectively (Table 6, middle panel). The association between the *H19* rs2839698 genotype and childhood leukemia was significant for survival time <5 years (adjusted OR = 1.43, 95%CI = 1.03–1.84), but not for those ≥5 years (Table 6, lower panel).

### 2.5. The Genotype–Phenotype Correlation of H19 rs2839698 Polymorphism

**Figure 1:**
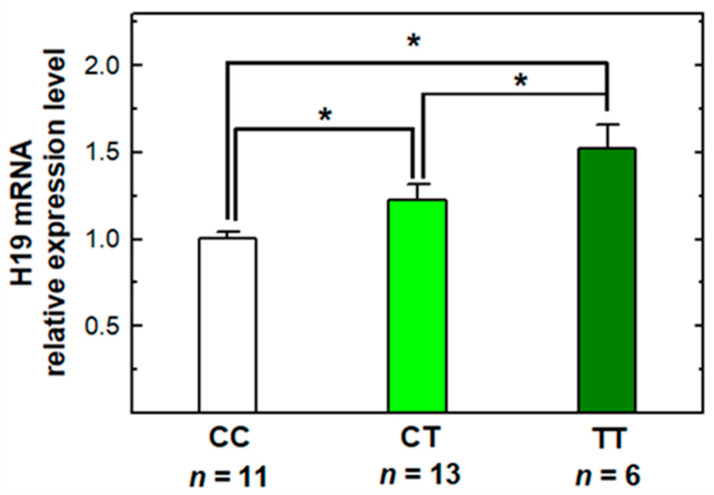
Comparison of H19 mRNA expression levels with different *H19* rs2839698 genotypes. Quantitative real-time polymerase chain reaction (RT-PCR) for three genotypes of *H19* rs2839698 from childhood leukemia patients was conducted and glyceraldehyde 3-phosphate dehydrogenase (GAPDH) was used as internal control. Fold changes were normalized by the GAPDH expression. * *p*-values *<* 0.05.

To investigate the genotype–phenotype correlation, we extracted 30 mRNA from the serum of childhood leukemia patients. These samples were obtained from the children before any chemotherapy. The frequencies of the *H19* rs2839698 CC, CT, and TT genotypes were 11 (36.7%), 13 (43.3%), and 6 (20.0%), respectively. The influences of various genotypes on the transcriptional expression of mRNA were evaluated by quantitative RT-PCR (Figure 1). As shown in Figure 1, the average level of mRNA for CT and TT genotypes of the *H19* rs2839698 was 1.22- and 1.52-fold, compared with that of the CC genotype, respectively. It is of statistically significantly higher level for patients with CT and TT genotypes than from those with the CC genotype (*p* < 0.0001) (Figure 1).

## 3. Discussion

Previous studies demonstrated that H19 was over-expressed in some types of tumors, such as hepatocellular carcinoma, lung, esophageal, and bladder cancer [28,29,30,31]. Therefore, *H19* has been suggested to play a role as an oncogene. The signaling network has not yet been fully established. Li and colleagues reported that H19 could bind directly to ISM1 and could also encode miR-675, which promotes gastric cell proliferation and metastasis by targeting *CALN1* [32]. H19 could also act as a molecular sponge for let-7, which is a well-known tumor suppressor miRNA capable of targeting and inhibiting oncogenic HMGA2, a mediator of epithelial–mesenchymal transition (EMT) in pancreatic ductal adenocarcinoma (PDAC). H19 inhibited let-7 and reversed its suppression on HMGA2, resulting in increased HMGA2-mediated EMT and metastasis in PDAC cells [33]. The “T” allele at *H19* rs2839698 was reported to be associated with bladder cancer [25], renal cell carcinoma [34], ovarian cancer [35], hepatoblastoma [36], hepatoma cell carcinoma [37,38], gastric cancer [39], colorectal cancer [40,41], and breast cancer [42]. Meanwhile, *H19* rs2839698 was reported not to be associated with oral cancer [43], lung cancer [44], cervical cancer [45], glioma [46], and neuroblastoma [47,48]. On the contrary, *H19* rs217717 was reported to be associated with altered risks of hepatoblastoma [36], gastric cancer [39], oral cancer [43], and lung cancer [44], while whether the “T” allele is a risk or protective is still controversial [26,42,49]. We have summarized the related literature in Table 7 and Table 8 for the genotypic findings for rs2839698 and rs217717 polymorphisms, respectively. There were some meta-analysis studies reporting that the “T” allele of rs2839698 is the risk allele for several types of cancer [50,51], while others reported null results [52,53,54,55]. Our current study is the first report of these two *H19* SNPs in childhood leukemia.

We found that individuals carrying the variant genotypes (CT and TT) and allele (T allele) of rs2839698 had significant increased risks of childhood leukemia in a Taiwan population (Table 2 and Table 3). In contrast, no such association was observed for rs217727 (Table 4 and Table 5). A previous genotype–phenotype correlation study showed that cancer-free controls carrying the variant genotypes (CT and TT) of *H19* rs2839698 had a higher expression of H19 mRNA in serum than those with the wild-type CC genotype among 80 healthy controls [39].

Our pilot study found CT and TT of *H19* rs2839698 had a higher expression of H19 mRNA in serum than those with CC genotype among 30 childhood leukemia patients (Figure 1). This is consistent with the findings among 74 gastric cancer patients [32]. More than that, the polymorphic variation of *H19* rs2839698 may be affecting the binding capacity between H19 and its target miRNAs. A previous study indicated that the oncogenic effect of H19 was partially mediated through the up-regulation of ISM1, a binding protein of H19 [32]. It is possible that the alterations in H19 structure via rs2839698 variation may affect the binding affinity of H19 to ISM1, which consequently promotes proliferation, migration, invasion, and metastasis [32]. However, the precise mechanisms of H19 action remain unclear, and further investigations are needed to verify the hypothesis.

The current study has a few limitations. First, the sample size was modest, and we could not perform stratified analyses. Second, since this was a hospital-based case-control study with all participants being recruited from the same hospital, there might be potential selection bias. However, the genotypic distribution in the control group was compatible with the Hardy–Weinberg expectations. Third, our study was conducted in a Taiwan population and our results need to be validated in other populations.

## 4. Materials and Methods

### 4.1. Recruitment of Childhood Leukemia and Control Participants

Childhood leukemia patients were identified and ascertained by a pediatric oncologist with pathologic confirmation. All basic and clinical characteristics of the recruited patients, including their histological details, were collected by physicians. All investigated subjects voluntarily participated this study, completed a questionnaire form with the help of their parents or guardians, and donated up to 5 mL blood. Healthy controls without prior history of any cancer were recruited through random sampling over the same period of 2005 to 2010 as we previously described [6,7,8]. Controls were matched to cases by age (±2 years) and gender. Finally, 532 participants (266 cases and 266 controls) under 18 years old were included in this study. All the participants were Taiwanese. This study was approved by the Institutional Review Board of China Medical University Hospital (DMR103-IRB-153, approved from 1 August 2018 to 31 July 2021).

### 4.2. DNA Extraction and Genotyping

Genomic DNA from the peripheral blood leukocytes was extracted (Qiagen, CA, USA). The genotypes of lncRNA *H19* rs2839698 and rs217727 were determined by polymerase chain reaction restriction fragment length polymorphism (PCR-RFLP) methodology. The PCR-RFLP genotyping methodology of *H19* rs2839698 and rs217727 polymorphisms were designed by the Terry Fox Cancer Research Lab. The contig near *H19* rs2839698 was amplified with forward primer 5′-AAG-GAG-CAC-CTT-GGA-CAT-CT-3′ and reverse primer 5′-CTG-CCA-CGT-CCT-GTA-ACC-AA-3′. The contig near *H19* rs217727 was amplified with forward primer 5′-GTC-GCT-ATC-TCT-AGG-TGA-AG-3′ and reverse primer 5′-GTG-GAG-GCT-TTG-AAT-CTC-TC-3′. DNA contig is amplified in a 25 µL reaction mixture containing 100 ng of genomic DNA of each subject, 20 µM of each primer, 5 µL of 10X PCR buffer with 1.5 mM MgCl_2_, and 1 unit of taq DNA polymerase. The PCR cycle was performed in a PCR Thermocycler (Bio-RAD, Hercules, CA, USA) using the following conditions: initial denaturation 94 °C for 5 min, followed by denaturation at 94 °C for 30 s, annealing at 64 °C for 40 s, and extension at 72 °C for 45 s. After completion of 40 PCR cycles, a final extension step was carried out at 72 °C for 10 min. The PCR amplicons were checked by 3% agarose gel electrophoresis. Then the *H19* rs2839698 and rs217727 PCR amplicons were digested by *Kas* I and *Eci* I, and the product sizes were verified with 3% agarose gel electrophoresis again. The *H19* rs2839698 product presented 3 different patterns: an intact single 226 bp fragment for the TT genotype; full-digested fragments of 106 and 120 bp for the homologous variant CC genotype; and fragments of 106, 120, and 226 bp for the heterozygous *variant* CT genotype, respectively. The *H19* rs217727 product presented 3 different patterns: an intact single 291 bp fragment for the TT genotype; full-digested fragments of 145 and 146 bp for the homologous variant CC genotype; and fragments of 145, 146, and 291 bp for the heterozygous variant CT genotype, respectively.

### 4.3. Quantitative RT-PCR Assay of H19 mRNA Expression

The transcriptional expression level of H19 was measured using RT-PCR analyzing RNA extracted from the serum of 30 childhood leukemia patients (Qiagen, Redwood, CA, USA). The sequences of H19 forward and reverse primers were 5-CCCACAACATGAAAGAAATGGTGC-3′ and 5-CACCTTCGAGAGCCGATTCC-3′, respectively. The sequences of internal control, GAPDH, were 5′-GAAATCCCATCACCATCTTCCAGG-3′ and 5′-GAGCCCCAGCCTTCTCCATG-3′. Real-time PCR was performed, and fold changes were normalized by the level of GAPDH. Each experiment was carried out blindly by two researchers and at least trice.

### 4.4. Statistical Analysis

The Student’s *t*-test was used to compare the age between the cases and controls. The Pearson’s chi-square test was used to examine Hardy–Weinberg equilibrium (HWE) in the controls and compare the distribution of *H19* rs2839698 and rs217727 genotypes between cases and controls. Logistic regression analyses were used to determine the associations between these two SNPs and childhood leukemia risks by calculating the odds ratios (OR) and 95% confidence intervals (CI). Age was adjusted in multivariate logistic regression analysis (Table 3 and Table 5). Any *p*-value less than 0.05 was considered statistically significant.

## 5. Conclusions

In conclusion, our results reported for the first time that the variant genotypes and alleles of *H19* rs2839698 were significantly associated with increased risks of childhood leukemia in Taiwan. Our study adds another piece of evidence that the H19 rs2839698 polymorphism may modulate the susceptibility to cancers. We also provided a summary of *H19* genotype associations with cancer risks. Further studies in all types of cancer across different populations are warranted to clarify the role of H19 polymorphisms in carcinogenesis.

## Figures and Tables

**Table 1:** Distribution of onset age and gender of the 266 childhood leukemia patients and 266 healthy controls.

Characteristics	Cases, *n* = 266			Controls, *n* = 266			*p*-Value
	*n*	%	Mean ± SD	*n*	%	Mean ± SD	
Onset age, year			7.0 ± 4.4			8.3 ± 4.8	0.6483 ^a^
Gender							1.0000 ^b^
Boy	148	55.6%		148	55.6%		
Girl	118	44.4%		118	44.4%		
While blood cell counts (10^9^/L)			54.3 ± 75.9			7.5 ± 2.0	<0.0001
t(1;19)	11	4.1%					
t(4;11)	4	1.5%					
t(9;22)	6	2.3%					
ETV6-RUNX1 ^c^	28	10.5%					
Immunophenotype							
B subtype	227	85.3%					
T subtype	39	14.7%					
Risk classification							
Standard risk	130	48.9%					
High risk	67	25.2%					
Very high risk	69	25.9%					
Survival time, year							
<5	69	25.9%					
≥5	197	74.1%					

Abbreviations: SD, standard deviation; Notes: ^a^ Based on Student’s *t-*test; ^b^ based on chi-square test without Yates’ correction; ^c^ available for only some of the patients.

**Table 2:** Distributions of *H19* rs2839698 genotypes between childhood leukemia and control groups.

Index	Genotype	Cases	Controls	*p*-Value	Crude OR (95%CI)
rs2839698	CC	91 (34.2%)	119 (44.7%)		1.00 (Ref)
	CT	131 (49.3%)	117 (44.0%)	0.0429 *	**1.46 (1.08–2.14)**
	TT	44 (16.5%)	30 (11.3%)	0.0169 *	**1.94 (1.15–3.31)**
	CT+TT	175 (65.8%)	147 (55.3%)	0.0130 *	**1.68 (1.12–2.23)**
*P*_trend_				0.0277 *	
*P*_HWE_				0.8781	

Abbreviations: OR, odds ratio; CI, confidence interval; Notes: *p*-values are calculated by chi-square without Yates’ correction; * Bold values show significance.

**Table 3:** Allelic frequencies for *H19* rs2839698 polymorphism among childhood leukemia and control groups.

Allele	Cases, *n* (%) (*n* = 532)	Controls, *n* (%) (*n* = 532)	Adjusted OR (95%CI) ^a^	*p-*Value ^b^
C	313 (58.8)	355 (66.7)	1.00 (Reference)	0.0077 *
T	219 (41.2)	177 (33.3)	**1.53 (1.13–1.79)**	

Abbreviations: OR, odds ratio; CI, confidence interval. ^a^ Data adjusted for age. ^b^ Based on chi-square test without Yates’ correction; * Bold values show significance.

**Table 4:** Distributions of *H19* rs217727 genotypes between childhood leukemia and control groups.

Index	Genotype	Cases	Controls	*p*-Value	Crude OR (95%CI)
rs217727	CC	111 (41.7%)	114 (42.9%)		1.00 (Ref)
	CT	120 (45.1%)	120 (45.1%)	0.8857	1.04 (0.80–1.41)
	TT	35 (13.2%)	32 (12.0%)	0.6763	1.10 (0.72–1.92)
	CT+TT	155 (58.3%)	152 (57.1%)	0.7923	1.06 (0.74–1.45)
*P*_trend_				0.9165	
*P*_HWE_				0.9610	

Abbreviations: OR, odds ratio; CI, confidence interval; Notes: *p* values are calculated by Chi-square without Yates’ correction.

**Table 5:** Distributions of *H19* rs217727 genotypes between childhood leukemia and control groups.

Allele	Cases, *n* (%) (*n* = 532)	Controls, *n* (%) (*n* = 532)	Adjusted OR (95%CI) ^a^	*p-*Value ^b^
C	342 (64.3)	348 (65.4)	1.00 (Reference)	0.7000
T	190 (35.7)	184 (34.6)	1.06 (0.84–1.39)	

Abbreviations: OR, odds ratio; CI, confidence interval. ^a^ Data adjusted for age. ^b^ Based on chi-square test without Yates’ correction.

**Table 6:** Association between the *H19* rs2839698 genotype with clinical index of childhood leukemia.

Index	CC		CT+TT		Adjusted OR
	*n*	%	*n*	%	(95%CI) ^a^
Immunophenotype					
B subtype	77	33.9	150	66.1	1.26 (0.91–1.38)
T subtype	14	35.9	25	64.1	1.22 (0.83–1.85)
Risk classification					
Standard risk	47	36.2	83	63.8	1.28 (0.92–1.45)
High risk	21	31.3	46	68.7	**1.46 (1.04–1.87)**
Very high risk	23	33.3	46	66.7	**1.38 (1.02–1.79)**
Survival time (years)					
<5	22	31.9	47	68.1	**1.43 (1.03–1.84)**
≥5	69	35.0	128	65.0	0.83 (0.64–1.23)

Abbreviations: OR, odds ratio; CI, confidence interval; ^a^ ORs, 95%CI were calculated after adjusting for age and gender status; Bold values show significance.

**Table 7:** The summary of genotypic findings for *H19* rs2839698 polymorphism among cancer risk.

Author	Year	Cancer	Case/Control, *n*	Population	Highlight Findings
Verhaegh	2008	Bladder cancer	177/204	Netherlands	CT but not TT genotype is associated with decreased bladder cancer risk	
Cao	2020	Renal cell carcinoma	1094/1027	China	TT but not CT genotype is associated with increased renal cell carcinoma risk	
Zhang	2020	Ovarian cancer	219/203	China	TT but not CT genotype is associated with increased ovarian cancer risk	
Tan	2021	Hepatoblastoma	213/958	China	TT but not CT genotype is associated with increased hepatoblastoma risk	
Wu	2019	Hepatocellular carcinoma	359/1190	Taiwan	CT but not TT genotype is associated with increased hepatocellular carcinoma risk	
Yang	2018	Hepatocellular carcinoma	472/472	China	CT plus TT genotype is associated with increased hepatocellular carcinoma risk	
Yang	2015	Gastric cancer	500/500	China	TT but not CT genotype is associated with increased gastric cancer risk	
Yu	2020	Colorectal cancer	315/441	China	CT plus TT genotype is associated with decreased colorectal cancer risk	
Li	2016	Colorectal cancer	1147/1203	China	TT but not CT genotype is associated with increased colorectal cancer risk	
Safari	2019	Breast cancer	111/130	Iron	CT and TT genotypes are associated with increased breast cancer risk	
Lin	2017	Breast cancer	1005/1020	China	No association	
Guo	2017	Oral cancer	362/741	China	No association	
Wang	2019	Lung cancer	564/1536	China	No association	
Huang	2019	Cervical cancer	235/325	Taiwan	No association	
Deng	2020	Glioma	605/1300	China	No association	
Li	2019	Neuroblastoma	700/1516	China	No association	
Hu	2019	Child neuroblastoma	393/812	China	No association	

**Table 8:** The summary of genotypic findings for *H19* rs217727 polymorphism among cancer risk.

Author	Year	Cancer	Case/Control, *n*	Population	Highlight Findings
Verhaegh	2008	Bladder cancer	177/204	Netherlands	No association
Cao	2020	Renal cell carcinoma	1094/1027	China	No association
Tan	2021	Hepatoblastoma	213/958	China	CT but not TT genotype is associated with decreased hepatoblastoma risk
Wu	2019	Hepatocellular carcinoma	359/1190	China	No Association
Yang	2015	Gastric cancer	500/500	China	TT but not CT genotype is associated with increased gastric cancer risk
Li	2016	Colorectal cancer	1147/1203	China	No association
Safari	2019	Breast cancer	111/130	Iron	CT and TT genotypes are associated with decreased breast cancer risk
Lin	2017	Breast cancer	1005/1020	China	CT and TT genotypes are associated with increased breast cancer risk
Xia	2016	Breast cancer	464/467	China	No association
Guo	2017	Oral cancer	362/741	China	TT but not CT genotype is associated with increased oral cancer risk
Wang	2019	Lung cancer	564/1536	China	TT but not CT genotype is associated with increased lung cancer risk
Huang	2019	Cervical cancer	235/325	Taiwan	No association
Cao	2020	Renal cell carcinoma	1027/1094	China	No association
Deng	2020	Glioma	605/1300	China	No association
Li	2019	Neuroblastoma	700/1516	China	No association
Hu	2019	Child neuroblastoma	393/812	China	No association

## Data Availability

The data presented in this study are available on request from the corresponding author.
